# Supplementary material for: Identifying repeat domains in large genomes
Source: Genome Biol. 2006 Jan 31;7(1):R7. doi: 10.1186/gb-2006-7-1-r7 (PMC1431705; doi:10.1186/gb-2006-7-1-r7)
Supplement: Additional File 1 — A zipped file of browsable HTML files with a complete list of the connected components in the repeat domain graph of human Repbase. [file gb-2006-7-1-r7-S1.gz › html/subgraphs/796.pdf]

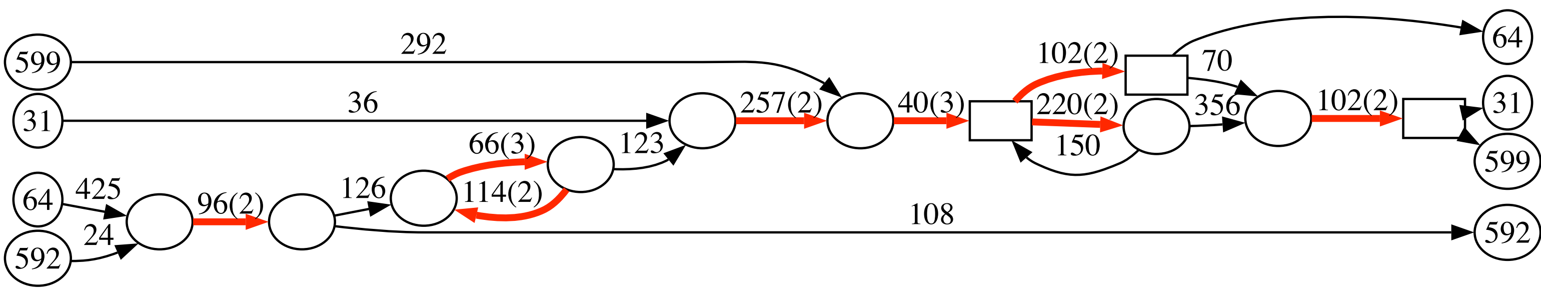
599
31
64
592
292
36
425
24
96(2)
126
66(3)
114(2)
123
257(2)
40(3)
102(2)
220(2)
150
70
356
102(2)
108
64
31
599
592
